# Prediction of mortality in critically-ill elderly trauma patients: a single centre retrospective observational study and comparison of the performance of trauma scores

**DOI:** 10.1186/s13049-020-00788-9

**Published:** 2020-09-23

**Authors:** Rebecca Egglestone, David Sparkes, Ahilanandan Dushianthan

**Affiliations:** 1 General Intensive Care Unit, University Hospital Southampton NHS Foundation Trust, Tremona Road, Southampton, SO16 6YD UK; 2 Acute Perioperative and Critical Care Group, Southampton NIHR Biomedical Research Centre, University Hospital Southampton/ University of Southampton, Tremona Road, Southampton, SO16 6YD UK

**Keywords:** Elderly, Trauma, Intensive care, Scoring, Outcome

## Abstract

**Background:**

Trauma in the elderly (≥ 65 years) population is increasing. This study compares the performance of trauma scoring systems in predicting 30-day mortality among the traumatised elderly patients admitted to the intensive care unit in a major trauma centre.

**Methods:**

We collected retrospective data for all elderly trauma patients admitted to our intensive care units between January 2012 and December 2017. We assessed Injury Severity Score (ISS), Geriatric Trauma Outcome Score (GTOS) and the Trauma Audit and Research Network’s (TARN) Probability of Survival (Ps17) between survivors and non-survivors. Receiver operator characteristic (ROC) curves were used to assess the performance of these scoring systems.

**Results:**

There were 255 elderly trauma patients with overall 30-day survival of 76%. There was a statistically significant difference in ISS, GTOS and Ps17 scores between survivors and non-survivors (*p* < 0.001). The area under the ROC curve (AUROC) was statistically significant for all 3, with AUROC of 0.66 (95% CI 0.59–0.74) for the ISS, 0.68 (95% CI 0.61–0.76) for the GTOS and 0.79 (95% CI 0.72–0.85) for the Ps17. The optimal cut-off points were ≥ 28, ≥ 142, ≤ 76.73 for ISS, GTOS and Ps17, respectively.

**Conclusion:**

Both ISS and GTOS scoring systems preformed equally in predicting 30-day mortality in traumatised elderly patients admitted to the intensive care unit, however neither were robust enough to utilise in clinical practise. The Ps17 performed more robustly, although was not developed for prognosticating on individual patients. Larger prospective studies are needed to validate these scoring systems in critically-ill elderly traumatised patients, which may help to facilitate early prognostication.

## Background

The population demographics of trauma patients are shifting, with admissions of increasingly older (≥ 65 years) and frail patients with multiple comorbidities [1]. As the population continues to age, this trend is set to continue. Older patients with severe injuries are at risk of more adverse outcomes when compared with younger patients, even where injury patterns are similar [2–5]. Critically-injured elderly trauma patients admitted to the intensive care unit (ICU) are particularly vulnerable with increased risk of hospital mortality or discharge to a supported care facility or nursing home [6].

Although generic intensive care-based scoring systems such as the Acute Physiology and Chronic Health Evaluation (APACHE) score are frequently used for risk stratification of critically ill patients, they are not specific for quantitative prognostication after severe traumatic injury. Several trauma-based scoring systems exist, such as the Injury Severity Score [7] (ISS), the Trauma and Injury Severity Score [8] (TRISS) and the more recent age-specific scoring system, the geriatric trauma outcome score (GTOS) [9]. However, none of these trauma scores have been tried and validated in the cohort of critically-ill, severely traumatised elderly patients.

We, therefore, performed a retrospective cohort study to investigate the outcome of all elderly trauma patients admitted to our intensive care unit and evaluated the performance of each scoring system in predicting mortality. Given the age-specific nature of the GTOS, we hypothesise that it would out-perform the TRISS and ISS scoring systems in our critically-ill, elderly patients. Furthermore, the Trauma Audit and Research Network (TARN) have developed a prediction model to calculate the probability of survival (Ps17) for all patients entered into the trauma database [10]. It has not been validated for prognostication in the individual patient, but it has been included to see how this prediction model performed, compared with the scoring systems. Appropriate early prognostication may help with patient and family centred goal-setting, enabling an objective clinical decision-making process to minimise suffering and reduce inappropriate interventions.

## Methods

This is a retrospective study of all trauma patients aged 65 years and above admitted to the University Hospital Southampton Hospital NHS Foundation Trust from January 2012 through to December 2017. This information was obtained from the trauma databases and included pre-calculated ISS and Ps17 scores. The Ps17 was the version of TARN’s probability of survival score in use during the study period. We were unable to calculate TRISS scores for many of our patients due to lack of relevant clinical details, especially physiological data, and as a result, we have not pursued the use of this score. Additional clinical details were obtained from all available electronic hospital databases. This study was part of a larger retrospective cohort study (CRIT-CO) and had the approval of local research and development (R&D) and National Health Research Authority (HRA).

GTOS was calculated using the formula: age + (2.5 x ISS) + 22 (if packed red blood cells (PRBCs) were given during the first 24 h). This value can then be converted to percentage mortality using the nomogram as published by the original authors [9], or using an online calculator [11]. The Ps17 estimates a probability of survival instead of the probability of mortality. Table 1 summarises the components of each of the scoring systems.

**Table 1:** Variable components of trauma scoring systems

Scoring system	Variable components of the scoring systems
GTOS	Age
	ISS
	Blood transfusion within 24 h
ISS	AIS score (1 - minor; 2 - moderate; 3 - serious; 4 - severe; 5 - critical; 6 - unsurvivable) given to 6 body regions (head, face, chest, abdomen, extremities and external)
TRISS	ISS
	SBP, respiratory rate, GCS
	Age (older or younger than 54)
	Blunt or penetrating mechanism
Ps17	ISS
	Age, Gender, GCS, Intubation
	Pre-existing medical conditions (Modified Charlson Index)

*AIS* abbreviated injury scale; *GCS* Glasgow coma scale; *GTOS* geriatric trauma outcome score; *ISS* injury severity score; *PRBCs* packed red blood cells; *Ps17* Probability of survival score; *TRISS* trauma injury severity score; *SBP* systolic blood pressure

Histograms for age, ISS, GTOS and Ps17 were all visually inspected and found not to be consistent with a normal distribution. Therefore, median and interquartile range (IQR) were used to describe the data, but mean and standard deviation have been included for completeness. Comparisons of age, ISS, GTOS and Ps17 of survivors vs non-survivors were carried out using the Mann-Whitney U test. Receiver operating characteristic (ROC) curves were then used to assess the performance of ISS, GTOS and the Ps17 for mortality prediction and age has been included for comparison. The optimal cut-off point for the prediction of mortality was obtained from the ROC curve using the Youden Index (Sensitivity + Specificity). Statistical analysis was carried out using MedCalc (version 19.4.0), SPSS (version 24) and Excel (version 16.36).

## Results

**Fig. 1:**
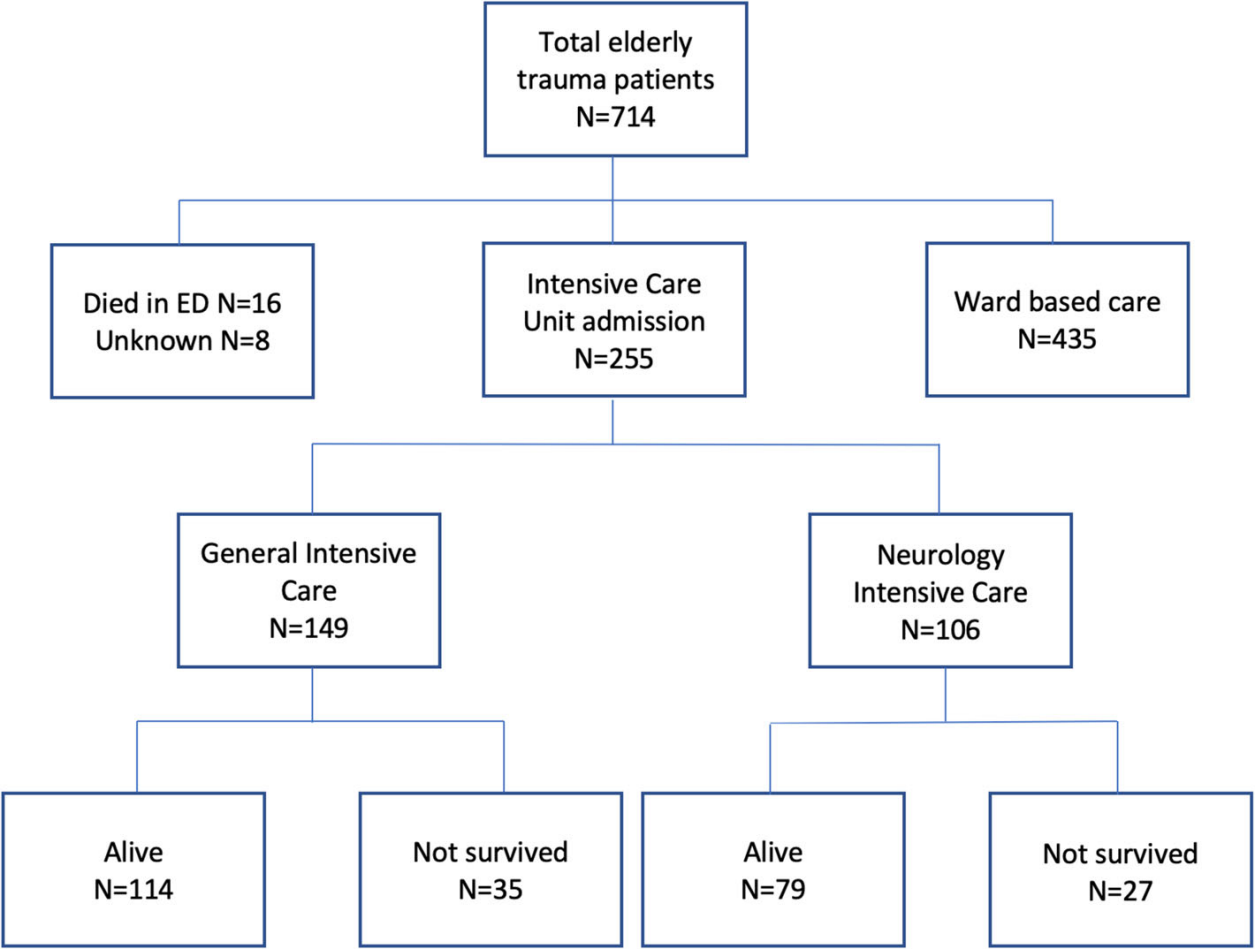
PRISMA flow-chart of patients included in the study

There were 714 elderly trauma patients hospitalised to our major trauma centre between January 2012 and December 2017. Of these, 255 required intensive care admission to either the general (GICU) or neuro-intensive care (NITU) units. Sixteen patients did not survive beyond the emergency department (Fig. 1).

Of those admitted to the intensive care units, 67% (*n* = 171) were male with a median age of 74 years (range 65–95 years). We divided these patients into two groups; 193 survivors (76%) and 62 non -survivors (24%), reflecting on the 30-day survival rate. The most common mechanism of injury was vehicular incident (54%), followed by fall from > 2 m (36%). The most commonly injured body region was head (40%), followed by multiple regions (19%). Most of these patients had medical comorbidities (86%), which included: hypertension (38%), ischaemic heart disease (29%), chronic obstructive pulmonary disease/asthma (18%) and diabetes mellitus (11%). A summary of patient demographics and injury mechanisms are presented in Table 2.

**Table 2:** Patient demographics and injury characteristics for survivors and non-survivors

Variable	All patients (***n*** = 255)	Survivors (***n*** = 193)	Non-survivors (***n*** = 62)
**Age**, years			
Median (IQR)	74 (69–80)	73 (69–79)	77 (71–84)
65–74	135 (52.9%)	109 (56.5%)	26 (41.9%)
75–84	90 (35.3%)	66 (34.2%)	24 (38.7%)
85–94	29 (11.4%)	17 (8.8%)	12 (19.4%)
> 95	1 (0.4%)	1 (0.5%)	0
**Gender**			
Female	84 (32.9%)	69 (35.8%)	15 (24.2%)
Male	171 (67.1%)	124 (64.2%)	47 (75.8%)
**Co-morbidity**			
Any	219 (85.9%)	167 (86.5%)	52 (83.9%)
Hypertension	98 (38.4%)	79 (40.9%)	19 (30.6%)
Heart disease	75 (29.4%)	59 (30.6%)	16 (25.8%)
Asthma/COPD	47 (18.4%)	32 (16.6%)	15 (24.2%)
Diabetes	28 (11.0%)	21 (10.9%)	7 (11.3%)
CVA/TIA	24 (9.4%)	18 (9.3%)	6 (9.7%)
**Mechanism of injury**			
Vehicle incident	138 (54.1%)	103 (53.4%)	35 (56.5%)
Fall > 2 m	92 (36.1%)	71 (36.8%)	21 (33.9%)
Stabbing	8 (3.1%)	7 (3.6%)	1 (1.6%)
Blow(s)	8 (3.1%)	7 (3.6%)	1 (1.6%)
Other	9 (3.5%)	5 (2.6%)	4 (6.5%)
**Most severely injured body region**			
Head	102 (40.0%)	72 (37.3%)	30 (48.4%)
Multiple	48 (18.8%)	38 (19.7%)	10 (16.1%)
Chest	46 (18.0%)	36 (18.7%)	10 (16.1%)
Limbs	23 (9.0%)	19 (9.8%)	4 (6.5%)
Spine	21 (8.2%)	18 (9.3%)	3 (4.8%)
Abdo	10 (3.9%)	9 (4.7%)	1 (1.6%)
Other	4 (1.6%)	0	4 (6.5%)
Face	1 (0.4%)	1 (0.5%)	0
**Length of stay**			
Median (IQR)			
Intensive care	5 (2–10)	5 (2–12)	2 (1–5)
Hospital	13 (6–24)	16 (9–30)	3.5 (1–8)

*COPD* Chronic obstructive pulmonary disease; *CVA* cerebrovascular accident; *IQR* interquartile range; *ISS* Injury Severity Score; *TIA* transient ischaemic attack

Outcome data was collected at hospital discharge, or 30-days if still an inpatient. ICU and 30-day survival rates were 88 and 76% respectively. Survivors tended to be younger with a lower ISS score and head injury rate. The median ICU and hospital stay for the survivors was 5 days (range 1–42) and 16 days (range 1–362) respectively. The median time to death from admission to ICU was 2 days and death following intensive care discharge was 1 day (range 1–12 days). Overall outcome data (mortality and neurological outcome defined by Glasgow Outcome Scale (GOS)) was known for 230 patients. Thirty-eight per cent had an unfavourable outcome, which included death or severe neurological disability and the rest had a favourable outcome, which is considered survival with a moderate neurological disability or good functional recovery according to the measured GOS at discharge. The discharge disposition in most cases was to another acute hospital (48%) followed by home (25%), rehabilitation facility (20%), nursing home (4%) and other institutes (3%).

**Fig. 2:**
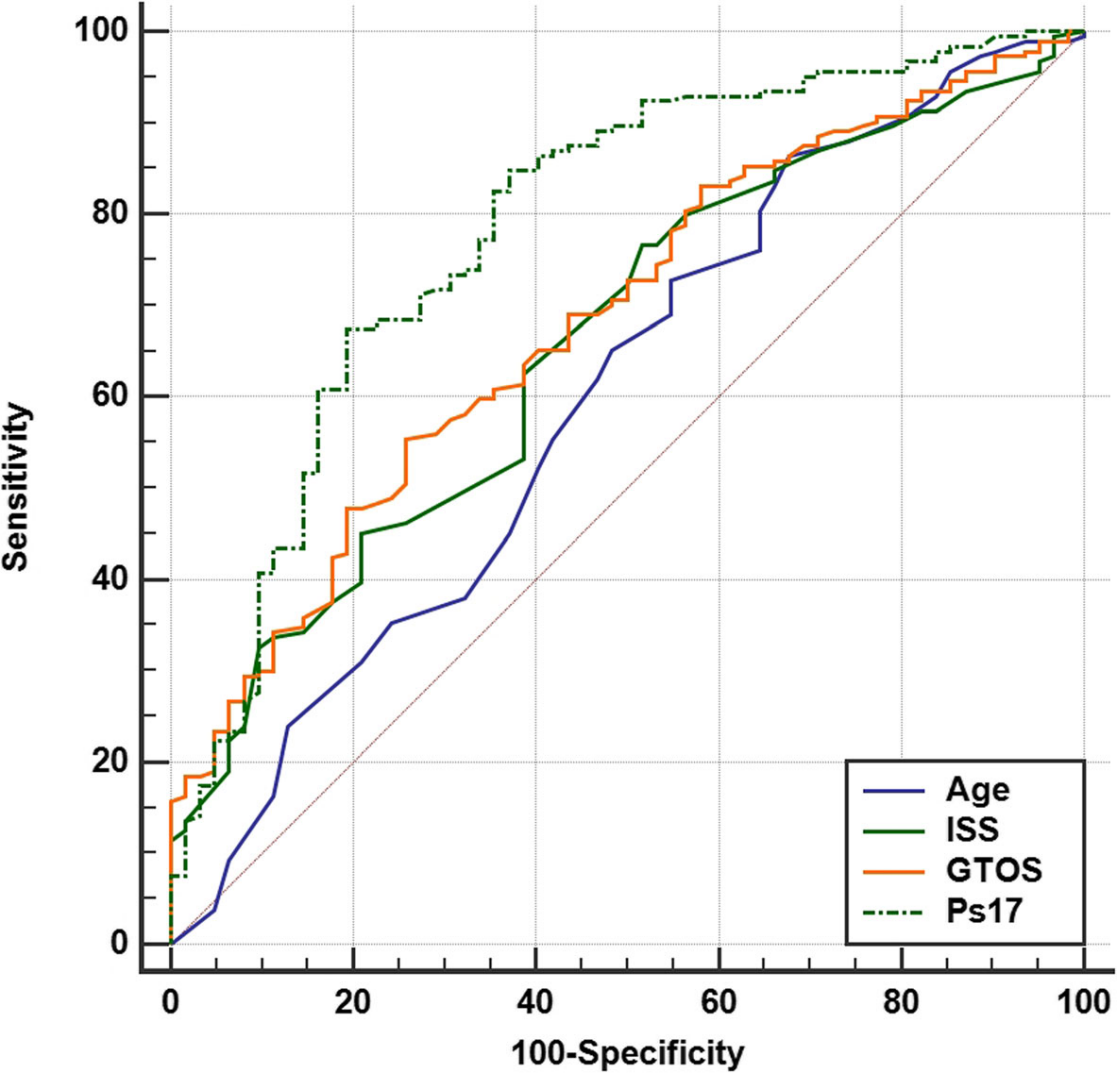
ROC curves of age, ISS, GTOS and Ps17 in mortality prediction of elderly trauma patients. ISS: Injury Severity Score; GTOS: Geriatric Trauma Outcome Score; Ps17: Probability of Survival; ROC receiver operator characteristic

**Fig. 3:**
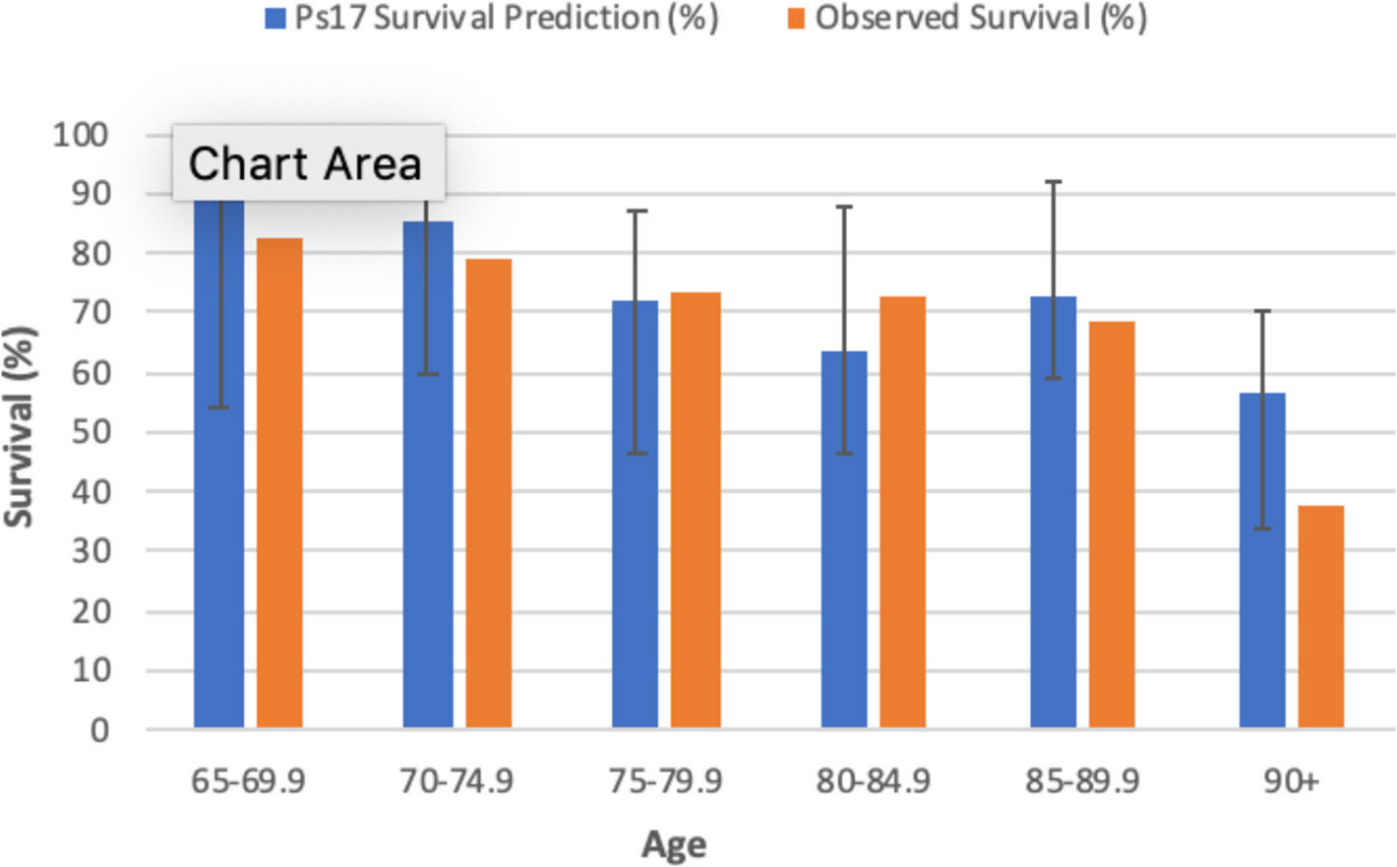
Comparison of observed 30-day survival and estimated Ps17 survival for age groups. Median survival vs observed survival in 5-year age ranges. Error bars reflect the IQR

We compared the ISS and GTOS scoring systems, and the Ps17 prediction model for their performance in predicting hospital mortality, in severely injured elderly patients admitted to the intensive care unit. Compared with survivors, the non-survivor group was older and had significantly higher ISS and GTOS scores, and significantly lower Ps17 scores (Table 3). The AUROC (Fig. 2) was statistically significant for all 3 of the scoring systems (Table 4). Age was a poor predictor of mortality, with an AUROC of 0.60 (95% confidence interval (CI) 0.536–0.662) (Fig. 2). Using the Ps17 we also compared predicted survival with our observed survival (Fig. 3). The observed survival rate closely matched the expected survival rate in all age ranges except in those aged ≥90 where observed survival was less than expected.

**Table 3:** Summary Statistics of Survivors and non-Survivors for Age, ISS, GTOS and Ps17 Scores

	Survivors (*n* = 193)	Non-survivors (*n* = 62)	Total (*n* = 255)	***P***-value (Mann-Whitney)
**Age, years**				
Mean ± SD	74.3 ± 6.7	77.3 ± 8.1	75.0 ± 7.14	0.014
Median, (IQR)	73 (69–79)	77 (71–84)	74 (69–80)	
**ISS**				
Mean ± SD	24.8 ± 11.96	31.7 ± 12.5	26.4 ± 12.4	0.000
Median, (IQR)	25 (17–30)	29.5 (24–38)	25 (17–34)	
**GTOS**				
Mean ± SD	141.7 ± 33.5	164.6 ± 35.6	147.2 ± 35.3	0.000
Median, (IQR)	139 (120–161)	158.5 (140–183)	143 (125–167)	
**Ps17**	(*n* = 185)	(*n* = 62)	(*n* = 247)	
Mean ± SD	76.4 ± 23.6	44.3 ± 31.3	68.3 ± 29.2	0.000
Median, (IQR)	85.67 (66–93)	40.04 (14–73)	81.29 (49–92)	

*ISS* Injury severity score; *GTOS* Geriatric trauma outcome score; *IQR* interquartile range; *Ps17* Probability of survival; *SD* standard deviation. *P* value reflects comparison between survivors and non-survivors

**Table 4:** Comparison of AUROC and cut-off values for ISS, GTOS and Ps17

Score	AUROC	95% CI	Specificity (%)	Sensitivity (%)	Cut-off
**ISS**	0.66	0.59–0.74	62.5	61.3	≥ 28
**GTOS**	0.68	0.61–0.76	54.9	74.2	≥ 142.25
**Ps17**	0.79	0.72–0.85	67.4	80.6	≤76.73

*AUROC* area under receiver operator curve; *CI* confidence interval; *ISS* injury severity score; *GTOS* elderly trauma outcome score; *Ps17* probability of survival

## Discussion

Severe trauma in elderly population imposes a significant health care burden and is associated with substantial morbidity and mortality [2, 3, 5]. Specific studies investigating the clinical outcomes of such patients in the intensive care setting are lacking. There were 255 trauma patients aged ≥ 65 years old admitted to our intensive care units, representing 36% of all elderly trauma patients admitted to our hospital during the 6 years. This proportion is slightly higher than the recently published United Kingdom (UK) National Trauma data, where just over a quarter of all trauma patients, regardless of their age, were admitted to either an ICU or high dependency facility [12]. However, our ICU admission rate was very similar to a large retrospective observational study of elderly trauma patients [13].

Intensive care unit outcomes of traumatic elderly patients are rarely reported. This study confirms that even within the defined elderly cohort, older age is associated with increased mortality. Each additional decade showed an increment in 30-day mortality, where ages 65–74, 75–84, ≥ 85 were associated with a mortality of 19, 29 and 40% respectively. Our overall ICU and 30-day mortality were 12 and 24% respectively. A larger multi-centre Spanish study of elderly trauma patients admitted to ICU with a median ISS score of 21, demonstrated an ICU mortality of 22 and 30% for patients aged 66–75 years and over 75 years, respectively [14]. Similarly, an American observational study of slightly younger patients with trauma (aged 55 and older) admitted to a surgical intensive care unit had a 28-day in-hospital mortality of 18% [15]. In general, our findings are comparable to those internationally published outcome data on elderly trauma ICU patients.

In our study, both the ISS and GTOS trauma scoring systems were predictive of mortality, with the AUROC being very similar at 0.66 and 0.68, respectively. Our AUROC for GTOS was much lower than the original publication and the follow-up validation study, where AUROC was 0.82 (95% CI 0.807–0.831) and 0.86 (95% CI 0.857–0.867), respectively [9, 16]. Several factors could account for this variation including differences in the patient population (general vs critically ill), validation sample size, and the availability of regional trauma services. By comparison, our patients were a subset of patients who were critically injured, with a higher ISS and had already survived from admission to intensive care. To the best of our knowledge, there are no other studies specifically looking at the use of scoring systems in this subset of elderly trauma patients.

The ISS is purely an anatomical scoring system [8], lacking the inclusion of physiological variables, age or underlying comorbidities, and although the AUROC was statistically significant, it lacked robust association. This finding is in line with a previous work, which has also demonstrated that the ability of the ISS to predict mortality in patients aged 65 and over is poor [17]. Therefore, neither the ISS or GTOS were robust enough to be helpful with ongoing individualised decision-making process in our intensive care setting. The Ps17, despite its better performance, with an AUROC of 0.79, is not designed as a prognosticative scoring system to guide decision making in individual patients. In our cohort, the Ps17 overestimated survival in the 90+ age range. This group included only 8 patients and is therefore too small to draw further conclusion as to why this was the case.

The limitations of this study include that this was an observational study relying on retrospective data collection from existing databases. Moreover, this study was conducted in a single major trauma centre and may not reflect the practices from other centres or be transferable to other trauma units worldwide. As a major trauma centre most of our patients were transferred from other areas. Consequently, we were not able to assess their longer-term mortality outcomes and discharge destination beyond 30 days. Given the retrospective nature of this study, our data set is not entirely exhaustive or comprehensive as some patients, despite their severity of trauma, may not have been admitted to the intensive care unit due to various reasons including prior established limitations of care. We had hoped to calculate the TRISS score to determine its utility in this population, however, the lack of complete physiological data set precluded us from doing so, with physiological variables most likely to be missing from the most critically ill. This problem is not unique to this dataset [18].

Larger, prospective, multicentre studies are needed to evaluate the utility of trauma scoring systems for the prognostication of elderly traumatised patients in the intensive care setting. Moreover, future studies should specifically focus on combination scoring systems to include organ specific measures and perhaps with the inclusion of frailty markers to assess outcome measures in this population. Besides, elderly trauma scores developed and validated in the USA may not be transferable universally, and an ICU specific UK based scoring system needs to be established. Furthermore, in addition to the short-term mortality outcomes, exploration of patient-centred outcomes such as quality of life, degree of functional and cognitive dependency and discharge destination may be of value.

## Conclusion

About a third of ≥ 65-year olds of all hospitalised trauma patients were admitted to an intensive care unit setting with a 30-day survival of 76%. Early prognostic prediction may help to support the clinical decision-making process. Both ISS and GTOS scores performed similarly but lacked robust association. Ps17 survival prediction model had a stronger association. Larger prospective studies are needed to establish robust scoring systems in this patient cohort and to evaluate, not only the mortality, but also ongoing quality of life associated with critically ill trauma in the elderly.

## Data Availability

The datasets used and/or analysed during the current study are available from the corresponding author on reasonable request.

## Abbreviations

AIS: Abbreviated injury scale
APACHE: Acute physiology and chronic health evaluation
AUROC: Area under receiver operating curve
CVA: Cerebrovascular accident
COPD: Chronic obstructive pulmonary disease
CI: Confidence interval
GICU: General intensive care
GTOS: Geriatric trauma outcome score
GCS: Glasgow coma scale
GOS: Glasgow outcome score
ISS: Injury severity score
ICU: Intensive care unit
IQR: Interquartile range
NITU: Neuro intensive care
PRBCs: Packed red blood cells
Ps17: Probability of survival
ROC curve: Receiver operator characteristic curve
SD: Standard deviation
SBP: Systolic blood pressure
TARN: The Trauma Audit and Research Network
TIA: Transient ischaemic attack
TRISS: Trauma injury severity score
UK: United Kingdom

## References

[1] The Trauma Audit and Research Network. Major trauma in older people. England and Wales, 2017. Available from: https://www.tarn.ac.uk/content/downloads/3793/Major%20Trauma%20in%20Older%20People%202017.pdf, Accessed 2 Jan 2019.

[2] Keller JM, Sciadini MF, Sinclair E, O'Toole RV (2012). Geriatric trauma: demographics, injuries, and mortality. J Orthop Trauma.

[3] Giannoudis PV, Harwood PJ, Court-Brown C, Pape HC (2009). Severe and multiple trauma in older patients; incidence and mortality. Injury..

[4] Caterino JM, Valasek T, Werman HA (2010). Identification of an age cut-off for increased mortality in patients with elderly trauma. Am J Emerg Med.

[5] Bruijns SR, Guly HR, Bouamra O (2013). The value of traditional vital signs, shock index, and age-based markers in predicting trauma mortality. J Trauma Acute Care Surg.

[6] Davidson GH, Hamlat CA, Rivara FP (2011). Long-term survival of adult trauma patients. JAMA..

[7] Baker SP, O'Neill B, Haddon W, Long WB (1974). The injury severity score: a method for describing patients with multiple injuries and evaluating emergency care. J Trauma.

[8] Boyd CR, Tolson MA, Copes WS (1987). Evaluating trauma care: the TRISS method. Trauma score and the injury severity score. J Trauma.

[9] Zhao FZ, Wolf SE, Nakonezny PA (2015). Estimating geriatric mortality after injury using age, injury severity, and performance of a transfusion: the geriatric trauma outcome score. J Palliat Med.

[10] The Trauma Audit and Research Network. The TARN Probability of Survival Model, August 2019. Available from: https://www.tarn.ac.uk/Content.aspx?c=3515. Accessed 8 May 2020.

[11] Palliate Consortium. GTOS and GTOS II prognosis calculator. Available from: http://palliateconsortium.com/develop/index.php, Accessed 19 Mar 2019.

[12] Moran CG, Lecky F, Bouamra O (2018). Changing the system – major trauma patients and their outcomes in the NHS (England) 2009–2017. EClin Med.

[13] Taylor MD, Tracy JK, Meyer W (2002). Trauma in the elderly: intensive care unit resource use and outcome. J Trauma.

[14] Penasco Y, Gonzalez-Castro A, Rodriguez Borregan JC (2017). Limitation of life-sustaining treatment in severe trauma in the elderly after admission to an intensive care unit. Med Int.

[15] McGreevy CM, Bryczkowski S, Pentakota SR (2017). Unmet palliative care needs in elderly trauma patients: can the palliative performance scale help close the gap?. Am J Surg.

[16] Cook AC, Joseph B, Inaba K (2016). Multicenter external validation of the geriatric trauma outcome score: a study by the prognostic assessment of life and limitations after trauma in the elderly (PALLIATE) consortium. J Trauma Acute Care Surg.

[17] Tamim H, Al Hazzouri AZ, Mahfoud Z (2008). The injury severity score or the new injury severity score for predicting mortality, intensive care unit admission and length of hospital stay: experience from a university hospital in a developing country. Injury..

[18] Bouamra O, Wrotchford A, Hollis S (2006). A new approach to outcome prediction in trauma: a comparison with the TRISS model. J Trauma.

